# Words to the Young

**Published:** 1875-07

**Authors:** 


### WORDS TO THE YOUNG.

We try to speak often and wisely to the young of both sexes, and we are often overjoyed to learn that they appreciate our efforts to interest them. We know that whatever our nation is to become, rests with the young; that the future of the country is in their hands. So we seek to make the boys and girls sweet, and pure, and moral, and thoughtful and intelligent, and find our reward in the fact that the work which we do in this direction will live long after we are gone.

There is not a man alive to-day, who, if he tell the truth, will not say that he has fairly wasted a good deal of time which he might have usefully employed. Young men and young women never or rarely reflect upon these things; but the reflection deepens as years advance. We wish then that we could live life over once more. If this were allowed us, how many errors would we avoid; how many follies would we surely escape; how many noble deeds would we do. At middle age we are just about ready to begin life in earnest. As we stand midway between the cradle and the grave, we begin to wonder and regret that we permitted the days of youth to go by only half enjoyed. For, the truth is, we do not really enjoy time which is misspent. We cannot really say that hours passed in idleness or dissipation are enjoyed. We are never really happy when we are not gaining something. There is, absolutely, no genuine comfort except in acquisition, and the supremest joy of all is found in the acquisition of useful knowledge.

Our young friends will find it vastly more agreeable to look back upon the accomplishment of some useful end than to take in by a backward glance, only time and opportunities thrown away. If every young man and young woman would determine that they will accomplish something in life, the world would move forward with wonderful strides towards all good and great ends. If each boy of 18 would say, "I will master a science or a business before I am 25," what a world we should have about us? Many a young man will read these words and will say: "I cannot be great. I cannot master anything. I do not know how to begin."

Let us tell you. Begin by mastering yourself. Begin by being superior to the ridicule of others. Begin by the fixed resolution—a resolution which you need not utter, but which you must act upon, nevertheless—that you will not smoke filthy tobacco, or put it in your mouth, or touch it, any more than you would any other dangerous and offensive thing. Then declare that you will never touch or taste certain other poisons, such as arsenic, brandy, strychnine, wine, prussic-acid, beer, and the like. Some of the poisons which we have named, you will not be likely to use; others you will be tempted to employ. The ones which you are tempted to use you should avoid more carefully than the rest. The ones which you are not likely to be urged to swallow, are no more dangerous than the more fashionable ones; but you are not likely to see them as often, and so will probably escape their injurious effects.

You may say that others use some of the poisons which we have spoken of, and live through it. Of course this is true; but you are a sensible young person—would you care to read this journal if you were not?—and you do not wish to imitate the dangerous practices of any man or woman. You have noticed that these poisons do not always kill, of course. This is merely because the dose taken has not been large enough. There is no disputing the fact that they are all poisons. All the opium and all the alcohol in the world, never yet made a single pound of solid, honest flesh.

A young man can do great things, in any direction, if he will but set about it with determination, and industry, and patience. No young man of any sort of character, will feel willing to be forever behind in the race for position, and honor and knowledge. He would prefer to lead and not to be led. If he desires to lead he can do so. It rests absolutely with him to determine the position which he is to occupy. Earnest endeavor and a right purpose, good habits, good morals and good health, clean hands and a pure heart—these are the essentials; With these all things are possible.

---

Weariness can sleep upon the flint when restive sloth finds the downy pillow hard.

A work of art is said to be perfect in proportion as it does not remind the spectator of the process by which it was created.

